# Meetings of Societies

**Published:** 1949-07

**Authors:** 


### Meetings of Societies

DISAPPOINTMENT has been expressed that the meetings of this or that Society are not reported. The Editor would remind secretaries and local correspondents that he is dependent on them for all such reports—and cannot publish what he does not receive. Papers already published elsewhere and lists of cases shown at clinical meetings are usually of little general interest.

### BRISTOL MEDICO-CHIRURGICAL SOCIETY

THE fifth meeting of the session was held in the University on Wednesday, 9th February, the President, Professor Drew-Smythe, in the Chair. Mr. V. B. Green-Armytage described his experiences in a paper entitled "**The Dramatic in Obstetrics and Gynæcology**" (p. 69), with lantern slide illustrations.

The sixth meeting was held on 9th March. Mr. J. A. Boycott gave an address on "**The Prophylactic Uses of Chemotherapy**" which we hope to publish in our next issue.

The seventh meeting on 13th April was attended by a number of local correspondents to the *Journal*. Mr. Geoffrey Keynes showed an excellent film of the operation of "**Thymectomy for Myasthenia Gravis**" and described the indications for and the results of the operation.

The eighth meeting of the session was held on 11th May. Dr. G. E. F. Sutton read a paper on "**Coronary Thrombosis,**" with special reference to the use of the electrocardiograph in diagnosis and prognosis.

### DEVON AND EXETER MEDICO-CHIRURGICAL SOCIETY

A CLINICAL meeting was held on Thursday, 21st April, 1949. Cases, specimens and X-rays were shown.

Cases and notes on cases were given by the following : (1) Mr. Darke and Mr. Lock, " **Spindle-celled Sarcoma of Groin** " ; (2) Mr. Greenberg and Dr. Fuller, " **Subacute Bacterial Endocarditis** " ; (3) Mr. Gairdiner, " **Multiple Gastric Ulcers** " ; (4) Mr. Daly, " **Five Cases of Meningitis** " ; (5) Mr. Greenberg and Dr. Seward, " **Anaemia due to Sepsis and ? Amyloid Disease** " ; (6) Mr. Scott, " **Four Cases of Mastoiditis** ".

### TORQUAY AND DISTRICT MEDICAL SOCIETY

THE Society's activities have been well maintained during the 1948–49 session. During the past year, six new members were elected, while six resigned. The membership at present stands at 124.

Speakers during the past year were : Dr. Charles Hill, Professor Chassar Moir, Dr. G. D. Kersley, Mr. M. F. Nicholls, F.R.C.S., Professor Alan Moncrieff, Mr. R. Belsey, F.R.C.S., Dr. D. W. D. Brooks.

The session commenced with a dinner and dance at the Grand Hotel, Torquay. Three interesting clinical discussions were held.

### PLYMOUTH MEDICAL SOCIETY

### PROGRAMME 1949–50

Oct. 5th (Greenbank, 8.30 p.m.). Annual General Meeting.

Oct. 26th, Dinner and Dance.

Nov. 11th and Dec. 16th (Greenbank, 8.30 p.m.). Clinical Meetings.

Nov. 25th, Mr. V. E. NEGUS : " **The Protection of the Air Passages** " with film.

### THE WEST COUNTRY PHYSICIANS' CLUB

THIS club met in October, 1948, at Cheltenham. Dr. C. Seward, " **The Clinical Approach to Anaemia** " ; Dr. W. A. Lister, " **Thrills and Swoons** " ; Professor A. V. Neale, " **Problems of Diabetes Mellitus in Childhood** " ; Dr. P. C. Gibson, " **Salicylic Acid in the treatment of Coronary Thrombosis** " ; Dr. R. F. Jarrett, " **Noma and Cancrum oris. Experience of 200 cases** ".

At Truro in May, 1949. Dr. C. J. Fuller, " **Symptomatology of Carcinoma of the Bronchus** " ; Dr. A. M. G. Campbell, " **Neurological Complications of Mumps** " ; Dr. R. G. M. Longridge, " **Intra-arterial injection of Histamine for Peripheral Vascular Disease** " ; Dr. C. R. Croft, " **Polycythaemia Vera** " ; Professor C. B. Perry, " **Acute Rheumatism as a Notifiable Disease** ".

The next meeting will be at Bath in October, 1949.

### THE SOCIETY OF ANAESTHETISTS

THE Society of Anaesthetists of the South-Western Region have held meetings at Taunton in April and in July at Plymouth. The third Annual General Meeting will be held in Bristol on 25th and 26th November when Dr. Beddard of Bath and Frome will be inaugurated as President. Details of the Bristol meeting can be obtained from the Hon. Secretary, Dr. G. L. Feneley, Avon Lodge, 8 Parry's Lane, Bristol 9.

### ASSOCIATION OF CLINICAL PATHOLOGISTS

### WESSEX BRANCH—*Secretary :* Dr. D. Woodman

MEETINGS have been held as under : November, 1948 : Canynge Hall, Bristol. 18th May, 1949 : Salisbury.

Programmes for next session : 26th November, 1949 : Dorchester. May, 1950 : Truro.

### BRITISH MEDICAL ASSOCIATION

### BATH, BRISTOL AND SOMERSET BRANCH

A MEETING of the above Branch was held on 16th February at the United Hospital, Bath. Professor Alexander Haddon gave an address on " **Recent Advances in our Knowledge of Neoplastic Growth** ".

A clinical meeting was held at the Bristol General Hospital on 23rd March.

### WILTSHIRE BRANCH

THE annual meeting was held at the County Mental Hospital, Devizes, on Sunday, 19th June. Mr. A. Duff was elected Chairman for 1949-50.

### BRISTOL DIVISION

ON 27th April, at the University, Sir Henry Cohen delivered a lecture on "**Recent Advances in Therapy** ".

The Annual General Meeting was held on Tuesday, 17th May.

*Executive Committee*, 1949–50

*Chairman :* Dr. W. Woolley. *Chairman Elect :* Dr. G. E. F. Sutton. *Hon. Secretaries :* Dr. H. M. Golding and Dr. W. H. Hayes. *Hon. Treasurer :* Dr. C. Dix.

*Representatives on Representative Body :* Dr. A. G. Heron, Dr. T. Milling, Dr. Winifred G. Nott, Dr. P. Phillips, Dr. W. Woolley. *Deputy Representatives :* Dr. W. H. Hayes, Dr. R. MacLean Courtney, Mr. F. J. Hector.

*Representatives of Public Health Officers :* Dr. A. A. Craig, Dr. R. C. Wofinden.

*Branch Council Representatives :* Professor R. J. Brocklehurst, Dr. G. L. Foss, Mr. F. J. Hector.

*Executive Committee :* Dr. D. M. Cameron, Dr. R. MacLean Courtney, Dr. W. R. Cole, Mr. A. G. Palin, Dr. H. K. V. Soltau.

*Public Relations Committee Secretary :* Dr. L. G. Robertson.

### BARNSTAPLE DIVISION

THE officers for the year are : Chairman, Dr. Valentine ; Vice-Chairman, Mr. Killard-Leavey ; Hon. Secretary, Dr. S. G. Brook ; Hon. Treasurer, Mr. K. G. W. Saunders ; Representative on the Branch Council, Dr. Wilson ; Vice-Representative on the Branch Council, Dr. S. L. Wake ; Representative at Annual Representative Meeting, Mr. Killard-Leavey ; Vice-Representative at Annual Representative Meeting, Dr. S. G.

Brook ; Executive Committee, Drs. Stewart and Ritchie together with the above officers.

Clinical meetings which had been abandoned during the war have been revived and the first Paper was given by Dr. Chas. Seward of Exeter following a Dinner. During the next year a programme is being arranged including showing of films and demonstration of cases. Another Dinner is being held in the autumn.

### WEST SOMERSET DIVISION

ON 17th March, Mr. Rodney Maingot gave a most interesting lecture on "**The Management of Patients with Gastric and Duodenal Ulcers**" ; and on 10th April, Dr. Tovey also gave a very interesting discourse on the Rh factors.

### TROWBRIDGE DIVISION

THE Trowbridge Division of the B.M.A. held its annual meeting on Wednesday, 25th May.

Dr. W. H. Royal was appointed Chairman for the ensuing year, and Dr. P. A. H. Rivett, Secretary.

The meeting was combined with a clinical meeting at Roundway Hospital, Devizes, at which an inspection of the special departments and selected wards of the hospital was undertaken, with a demonstration of electro-convulsive therapy.

### SALISBURY DIVISION

THE Annual General Meeting was held on Sunday, 29th May : the following were appointed for the ensuing year. Chairman, Mr. A. Duff ; Vice-Chairman, Dr. P. D. Abbatt ; Representative, Dr. G. Newton Dunn; Secretary-Treasurer, Dr. G. W. D. Henderson ; Executive Committee, Dr. Greenstreet, Dr. Whitehead, senr.

